# TET1-mediated DNA hydroxymethylation activates inhibitors of the Wnt/β-catenin signaling pathway to suppress EMT in pancreatic tumor cells

**DOI:** 10.1186/s13046-019-1334-5

**Published:** 2019-08-09

**Authors:** Jian Wu, Hongzhe Li, Minmin Shi, Youwei Zhu, Yang Ma, Yiming Zhong, Cheng Xiong, Hao Chen, Chenghong Peng

**Affiliations:** 1 Department of General Surgery, Ruijin Hospital, School of Medicine, Shanghai Jiao Tong University, Shanghai, China; 2 Research Institute of Digestive Surgery, Ruijin Hospital, School of Medicine, Shanghai Jiao Tong University, Shanghai, China

**Keywords:** TET1, DNA methylation, Pancreatic tumor, Epithelial-mesenchymal transition, Wnt/β-catenin pathway

## Abstract

**Background:**

Ten-eleven translocation 1 (TET1) is a dioxygenase that converts 5-methylcytosine (5-mC) to 5-hydroxymethylcytosine (5-hmC) to induce DNA demethylation. TET1 has been reported to be absent in cancers, and to influence various oncogenes and anti-oncogenes. However the function of TET1 in pancreatic tumor remains poorly understood. In this study, we investigated the role of TET1 in the progression of pancreatic tumor and its mechanism of tumor suppression.

**Methods:**

Quantitative real-time PCR (qRT-PCR), immunohistochemical (IHC) staining and dot blot were performed to detect the TET1 and 5-hmC expression in pancreatic tumor tissues and its adjacent non-tumor tissues. The clinical parameters significance of pancreatic tumor tissues was determined statistically. TET1 over-expression and knock-out cell lines were built and confirmed in vitro. Cell proliferation assay, wound-healing assays, transwell migration assay and nude mice model of orthotopic pancreatic cancer implantation were performed to assess the function of TET1 in pancreatic tumor. Western blot, qRT-PCR, immunofluorescence (IF), bisulfate sequencing (BSP), Chromatin immunoprecipitation (ChIP) were used to uncover the mechanism.

**Results:**

TET1 levels and 5-hmC content were downregulated in pancreatic tumor tissues and cell lines, and pancreatic tumor patients with low TET1 levels had a shorter overall survival than patients with high levels of TET1. TET1 suppressed pancreatic tumor proliferation and metastasis in vivo and in vitro. TET1 bound to the secreted frizzled-related protein 2 (SFRP2) promoter and catalyzed demethylation to activate transcription of SFRP2, inhibiting both the canonical and non-canonical Wnt signaling pathways, and ultimately obstructing epithelial-mesenchymal transition (EMT) in pancreatic tumors.

**Conclusion:**

We found TET1 plays as a suppressor in pancreatic tumor progression via obstructing Wnt signaling pathways.

**Electronic supplementary material:**

The online version of this article (10.1186/s13046-019-1334-5) contains supplementary material, which is available to authorized users.

## Background

DNA methylation is an important mechanism of epigenetics, which alters gene expression without changing the sequence of DNA. The members of the ten-eleven translocation family (TET1, TET2, and TET3) of dioxygenases play a key role in DNA demethylation by catalyzing the hydroxylation of 5-methylcytosine (5-mC) to 5-hydroxymethylcytosine (5-hmC) [1–3] and regulating the methylation levels of 5-cytosine (5-C) in coordination with DNA methyltransferases (DNMTs) [4]. Enrichment of 5-hmC and decrease of 5-mC in promotor CpG islands are considered to be indicators of gene activation [5]. The 5-hmC content in CpG islands is mainly regulated by the TET family [1, 3]. Abnormal expression or gene mutations of members of the TET family have been reported in various kinds of cancers in humans. It was reported that aberrant expression of TET1 was more frequent in solid tumors [6–8], *TET2* was frequently mutated in hematopoietic tumors [9], while TET3 remains less discussed [10].

TET1 was first found in a patient with a rare variation of t (10, 11)(q22;q23) acute myeloid leukemia (AML), and the gene is located at chromosome 10q21.3 [11]. With the exception of aberrant expression in cancer progression, TET1 is involved in cell differentiation [12, 13]. The role of TET1 in gastric [6], breast [14], hepatic [7], prostate [8], and colon [15] cancer is well-studied. In these tumors, TET1 is mostly considered to be a tumor suppressor, while it is also reported as an oncogene [16]; it binds directly to the promoters of anti-oncogenes, catalyzes 5-mC hydroxylation to initiate demethylation, and induces target genes involved in transcriptional activation, such as phosphatase and tensin homolog (PTEN) in gastric cancer [6].

Pancreatic tumors are among the most lethal malignant tumors. Unlike the steady increase in survival rates for most cancers, the 5-year survival rate of patients with pancreatic tumors is 8%; furthermore, the 5-year survival rate for patients diagnosed with advanced stages of pancreatic cancer is only 2% [17]. Aberrant DNA methylation in pancreatic tumors has been widely studied in recent years [18, 19], while the relationship between TET1 and pancreatic cancer is still unclear, with only a decrease in 5-hmC levels reported by Yang [20]. In this study, we determined the potential involvement of TET1 in pancreatic cancer progression, focusing on tumor proliferation and metastasis.

We found TET1 was downregulated in pancreatic tumor tissue compared to adjacent non-tumor tissue in > 3/5 pancreatic cancer patients, and low expression of TET1 was associated with shorter overall survival. We utilized RNA arrays to demonstrate that overexpression of TET1 inhibited pancreatic tumor epithelial-mesenchymal transition (EMT) by suppressing the Wnt/β-catenin signaling pathway, but not the Transforming growth factor (TGFβ) or NOTCH signaling pathways. Secreted frizzled-related protein 2 (SFRP2), the upstream inhibitor of the Wnt/β-catenin pathway, was transcriptionally activated by overexpression of TET1, which increased 5-hmC content in the SFRP2 promoter. We also revealed that TET1 inhibited proliferation of pancreatic tumors by inducing GO/G1 arrest in the cell cycle, although the underlying mechanism requires further study. This is the first report of TET1 as a suppressor of pancreatic tumors, further elucidating the role of TET1 in pancreatic cancer.

## Materials and methods

### Pancreatic tumor cell lines and tissue samples

Cell lines were obtained from Cell Bank, Shanghai Institutes for Biological Sciences, Chinese Academy of Sciences, Shanghai, China. Sw1990 and BXPC3 cells were cultured in Dulbecco’s Modified Eagle’s Medium and RPMI 1640 (Gibco, Thermo Fisher Scientific, Waltham, MA, USA), respectively, supplemented with 10% fetal bovine serum (FBS, Biological Industries, Beit-Haemek, Israel), 100 IU/mL penicillin, and 100 μg/mL streptomycin (Gibco) at 37 °C in humidified air with 5% CO_2_. A total of 15 paired pancreatic tumor and adjacent non-tumor tissue samples were collected from Ruijin Hospital, School of Medicine, Shanghai Jiao Tong University, Shanghai, China. The pancreatic tissue microarray was purchased from Shanghai Outdo Biotech, Shanghai, China. All patients included in the analysis were treated by R0 resection, and didn’t received radiotherapy, chemotherapy and neoadjuvant therapy.

### Plasmids and stable cell lines

The full-length human TET1 overexpression plasmid (TET1-OE) was obtained from Addgene (Cambridge, MA, USA). We also constructed a TET1-CD-mutated plasmid (TET-MUT) by modifying amino acids 1672 H to Y and 1674 D to A. The vector backbone pEF1a (Invitrogen, Carlsbad, CA, USA) was set as the empty vector (EV). The plasmids were transiently transfected into the SW1990 and BXPC-3 cell lines with Lipofectamine 2000 (Invitrogen) following the manufacturer’s protocol.

Stable TET1 knockout cell line SW1990-KO was constructed by CRISPR/CAS9. The lentivirus packed with pGMLV-CA1 expressing Caspase-9 and lentivirus packed with pGMLV-GM2 expressing gRNA targeting the sequence 5′-CAGCACGCATGAATTTGGAT-3′ in the TET1 sequence were constructed by Genomeditech, Shanghai, China. We conducted selection of the monoclonal TET1 knockout cell line in our laboratory. We also built a SW1990 control cell line SW1990-NC cell line by transfected SW1990 only with the lentivirus packed with pGMLV-CA1 expressing Caspase-9.

The stable TET1 overexpressing cell line BX-TET1-OE was generated using the dCAS9-SAM system. Lentiviruses expressing dCAS-VP64, MS2-P65-HSF1, and gRNA, respectively were constructed by Genomeditech. The TET1 overexpressing gRNA was designed to target the sequence 5′-AGGGGGTCGAGAGGGAGTCG-3′. We also built a BXPC-3 control cell line BX-TET1-NC cell line by transfected BXPC-3 with the dCAS9-SAM system, which gRNA was designed to target the sequence 5′- GTTCTCCGAACGTGTCACGT-3′.

SFRP2 siRNA was designed as follows: Si-SFRP2–1 5′-GTGAGGAGATGAACGACAT-3′, Si-SFRP2–2 5′- GCAAGACCATTTACAAGCT-3′, Si-SFRP2–3 5′- GCATCGAATACCAGAACAT-3′.

### RNA extraction and RT-PCR analysis

Total RNA was extracted with TRIzol® (Invitrogen) and reverse-transcribed using a cDNA reverse transcription kit (Toyobo Life Science, Shanghai, China). The cDNA was diluted 1:10 in DNase and RNase-free dH_2_O. Real-time PCR was performed with an ABI 7900 instrument using SYBR Green PCR Master Mix (Toyobo Life Science). Quantification was determined by the delta-delta Ct method, expressed in arbitrary units, and normalized to GAPDH. The primers used are listed in Additional file 1: Table S1.

### DNA extraction and dot blot

Genomic DNA of tissues and cell lines was extracted using a DNA isolation kit (Tiangen Biotech, Beijing, China). The DNA was denatured at 99 °C for 5 min, then cooled on ice. Subsequently, DNA was spotted onto a nitrocellulose membrane and air-dried. The DNA was UV-cross-linked to the membrane, which was then blocked in 5% non-fat milk in TBST, incubated with anti-5-hmC or anti-5-mC antibody (GeneTex, Irvine, CA, USA) at 4 °C overnight. The membrane was then incubated with anti-mouse IgG-HRP secondary antibody. Finally, the membrane was visualized by ECL (Beyotime Biotechnology, Shanghai, China).

### Protein extraction and western blotting

Total protein was extracted with RIPA buffer containing PMSF and sonicated. Nuclear protein and cytoplasmic protein were extracted with the Nuclear-Cytosol Extraction Kit (Beyotime Biotechnology). Protein samples were separated by SDS-PAGE and transferred onto PVDF membrane and blocked with 5% BSA in TBST, washed with TBST, incubated with the primary antibody at 4 °C overnight. The membrane was washed and incubated with HRP-conjugated secondary antibody at room temperature for 2 h. The membrane was washed again and visualized by ECL. TET1 antibodies were purchased from GeneTex. GAPDH, GSK-3β, phospho-GSK-3β (Ser9), p16, and EMT antibodies were purchased from Cell Signaling Technology (Danvers, MA, USA).

### Colony formation assay

BXPC-3 cells were cultured in 6-well plates and transfected with 5 μg of TET1-OE, TET1-Mut, or EV controls using Lipofectamine 2000 according to the manufacturer’s protocol. After 48 h, cells were harvested and seeded at 2000 cells/well in 6-well plates for 14 days. Colonies were then stained with gentian violet (ICM Pharma, Singapore) and counted.

### Cell proliferation assay

The transiently transfected BXPC-3, SW1990-KO, and SW1990-NC cells were seeded at 2000 cells/well in 96-well plates. Proliferation was detected with a Cell Counting Kit-8 (Dojindo, Shanghai, China) after 24, 48, and 72 h, measured by OD450.

### Wound healing and transwell migration assays

The 3 transiently transfected BXPC-3 cell lines, SW1990-KO, and SW1990-NC were cultured in 6-well plates until confluent. The monolayers were scratched, washed with PBS, cultured in serum-free RPMI 1640, and viewed after 0, 24, and 48 h.

For the transwell migration assay, we used Corning Transwell chambers (Corning Life Sciences, Corning, NY, USA). For migration, 2 × 10^5^ cells suspended in 200 μL serum-free medium were added to the upper chamber with 400 μL medium containing 10% FBS added to lower chamber, and the chambers were cultured for 24 h. For invasion, the upper chamber was coated with 20 μg Matrigel (BD Biosciences, San Jose, CA, USA) and cultured for 48 h using the protocol described above. Subsequently, the cells that migrated through the membrane were fixed and stained with crystal violet, and counted in 3 random fields.

### Flow cytometry analysis

Stable TET1-overexpressing BX-TET1-OE, knockout SW1990-KO, and their negative control cell lines were cultured in 6-well plates until 70% confluent and starved overnight. RPMI 1640 containing 10% FBS was added to the wells. After 6 h, the cells were harvested and fixed in ice-cold 70% ethanol overnight at 4 °C, and mixed with propidium iodide (BD Pharmingen, BD Biosciences) for 30 min at 37 °C in the dark. Data were analyzed with CellQuest software (BD Biosciences).

### Immunohistochemical staining (IHC)

The tissue samples and a tissue microarray including 63 pancreatic tumor tissues (57 of 63 had their adjacent non-tumor tissues) purchased from Shanghai Outdo Biotech were collected for IHC staining. The tissue samples of tumor xenograft mice were collected three regions per tumor. The tissue sections were de-waxed, hydrated, incubated in citrate buffer for antigen retrieval and fixed, then blocked in TBST containing 5% BSA. Tissue sections were incubated with anti-TET1 antibody at 4 °C overnight, washed in TBST 3 times for 10 min and incubated with anti-mouse IgG-HRP secondary antibody for 2 h at room temperature, then washed again in TBST.

### Immunofluorescence staining

BX-TET1-OE and BX-TET1-NC cells were incubated on coverslips in 6-well plates, and some were transfected with siRNA siSFRP2 for 48 h. Cells were then fixed in 4% paraformaldehyde and incubated with β-catenin primary antibody at 4 °C overnight and fluorochrome-labeled anti-rabbit IgG secondary antibodies. Cells were then stained with DAPI and subsequently viewed by confocal microscopy.

### RNA array analysis

EMT RT^2^ Profiler PCR Arrays were purchased from Qiagen (Hilden, Germany). RNA extracted from BX-TET1-OE, BX-TET1-NC, SW1990-KO and SW1990-NC cell lines were reverse-transcribed into cDNA and added to the RT^2^ Profiler PCR Array plates following the manufacturer’s protocol. The results were analyzed by Qiagen’s online tool (https://www.qiagen.com/).

### GlucMS-qPCR to determine 5-hmC and 5-mC content

We performed GlucMS-qPCR to detect 5-hmC and 5-mC content in promoter regions using the EpiMark® 5-hmC and 5-mC Analysis Kit (New England Biolabs, Hitchen, UK). Genomic DNA was treated with T4 Phage β-glucosyltransferase and UDP-Glucose to modified 5-hmC sites to 5-ghmC, and then digested by *Msp*I and *Hap*II following the manufacturer’s protocol. *Msp*I cleaves 5-hmC and 5-mC, but not 5-ghmC, while *Hap*II cleaves 5-hmC, 5-mC, and 5-ghmc. Then, we analyzed the enzyme-digested product by semi-quantitative PCR. The primers used to determine 5-mC and 5-hmC content in the promoter of SFRP2 were: forward 5′-TAGGATTTCTTTAAACAACAAACAGAGAA-3′ and reverse 5′-ATGCCTGGCAACCCAGCAGAAACT-3′.

### Bisulfate sequencing

Genomic DNA was bisulfate-treated using the EpiTect Bisulfite Kit Qiagen following the manufacturer’s protocol. Bisulfate-treated DNA was amplified by PCR with the following primers: SFRP2 Forward: 5′- TTTTTTACGGTATTGGGGAGTATAT-3′ and Reverse: 5′-CCGAAATTTCTACTAAATTACCAAAC-3′. PCR products were then cloned into the T-Easy Vector (Quanshijin Biotechnology, Beijing, China). For each treated group, 10 clones were randomly selected for sequencing, and the results were analyzed by QUMA, an online CpG methylation analysis tool (http://quma.cdb.riken.jp/).

### Chromatin immunoprecipitation (ChIP)

ChIP was performed in BXPC-3 cells. Cells cultured in 10 cm plates were cross-linked with formaldehyde and processed following the ChIP kit protocol (EMD Millipore, Burlington, MA, USA). The target DNA fragments were detected by PCR. The primers used to detect target DNA fragments are as follows: SFRP2-Forward 5′-AAACAGAGAAGCCTGGCCG-3′, SFRP2-Reverse 5′-TTCGGACTGGGGCAAAACAA-3′.

### Animal model of tumor xenograft

BALB/c nude mice (6-weeks old, male) were purchased from the Chinese Academy of Sciences.

(Shanghai, China) and kept in a specific pathogen-free environment.

For subcutaneous tumorigenesis, BX-TET1-OE and negative control cell line BX-TET1-NC were harvested and suspended in 1 × PBS. A total of 3 × 10^6^ cells were injected into mice subcutaneously.

For in vivo orthotopic implantation of the pancreatic cancer model, BX-TET1-OE and BX-TET1-NC cells were labeled with firefly luciferase, with 1 group of BX-TET1-OE being transfected with siSFRP2 for 48 h before injection. Then 3 × 10^6^ cells suspended in 100 μL medium (containing 1/3 Matrigel) were injected into the pancreata of mice. An additional abdominal injection of 200 nmol/kg si-SFRP2 was performed in si-SFRP2 group after 1 week, while 200 nmol/kg si-NC were injected into other two groups. Metastasis was photographed using a Xenogen noninvasive bioluminescence In Vivo Imaging System (IVIS, PerkinElmer, Waltham, MA, USA) as described previously [21].

Mice were euthanized after 3 weeks. The implanted tumors and metastatic foci were observed by general observation, H&E staining, and IHC staining. The pancreatic tumor and liver metastasis samples of tumor xenograft mice were collected three regions per tumor and liver.

### Statistics

Data analysis was performed using SPSS 19.0 software (SPSS Inc., Chicago, IL, USA), and data are presented as the mean ± SD from at least 3 separate experiments. Kaplan–Meier method was used for overall survival. Student’s *t*-test, Kendall’s tau-b, and Spearman correlations were also used. In Kendall’s tau-b correlation analysis, the dichotomous variables: 1, − 1 were used instead of levels of 5-hmC, TET1, TET2, TET3 in tumor tissues higher in non-tumor tissues or the opposite, respectively. Statistical significance was set as *P* < 0.05.

## Results

### TET1 is downregulated in pancreatic tumors and correlates with levels of 5-hmC

**Fig. 1:**
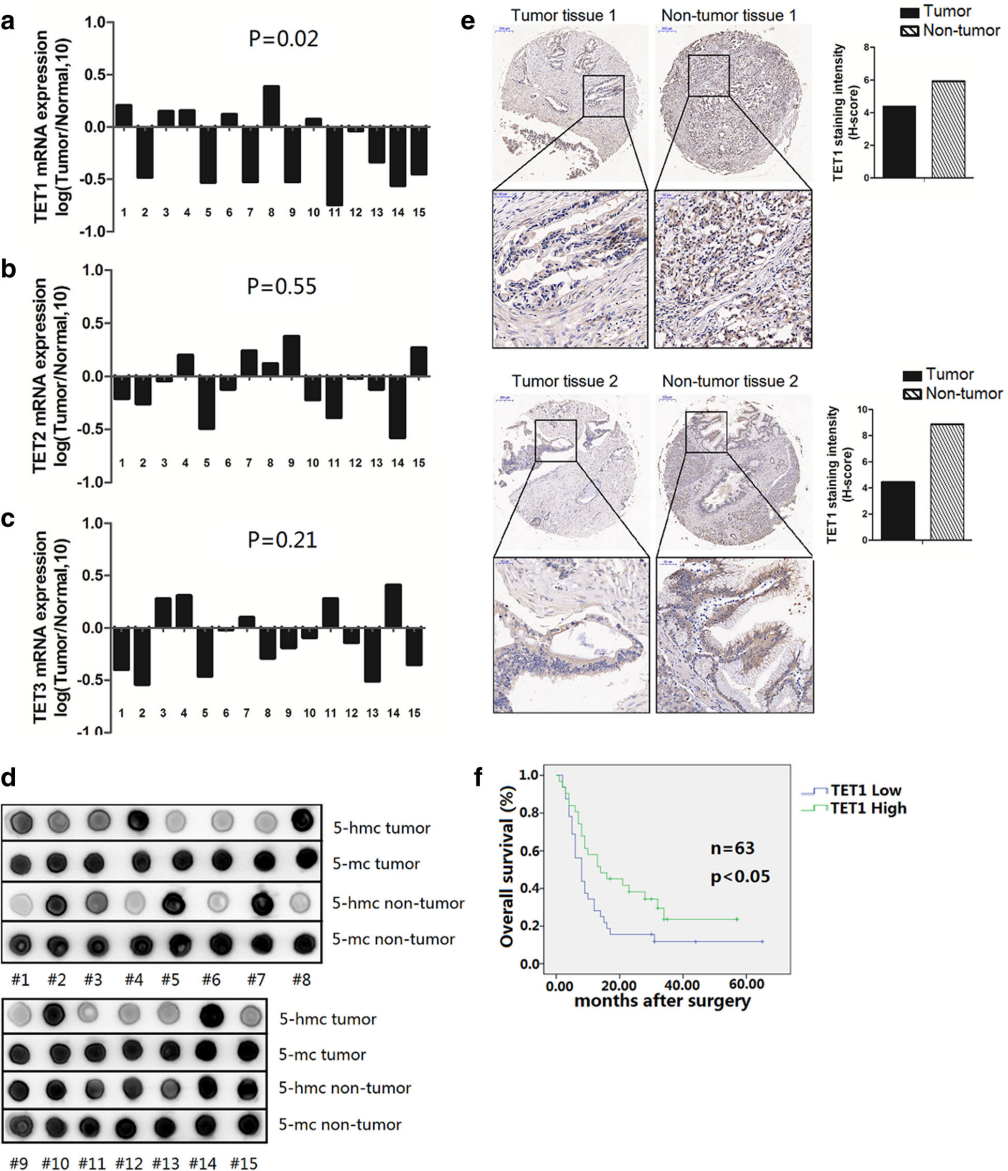
TET1 is downregulated in pancreatic tumors. **a** TET1, **b** TET2, and **c** TET3 mRNA levels in pancreatic tumor tissues verses adjacent non-tumor tissues. Data are shown as log2-fold changes (tumor/normal). Results were analyzed by Student’s *t*-test. **d** 5-hmC and 5-mC levels in pancreatic tumor tissues verses adjacent non-tumor tissues were detected by dot blot. **e** IHC analysis of TET1 in pancreatic tumor tissues and adjacent non-tumor tissues (Original magnification, 20×. Enlarged graph, 80×). **f** Kaplan–Meier analysis of overall survival in 63 patients with pancreatic cancer

To explore the levels of members of the TET family and their hydroxymethylation in pancreatic tumors, we analyzed 15 paired tumor and adjacent non-tumor tissues. We found that TET1 mRNA but not TET2 or TET3 was significantly reduced in tumor tissues compared with non-tumor tissues (*P* = 0.02, *P* = 0.55, and *P* = 0.21 respectively) (Fig. 1a–c). To confirm the influence of the TET family in hydroxymethylation, we also determined the 5-hmC and 5-mC content in paired tissues. In accordance with the results reported by Yang [20], dot blot analysis indicated the level of 5-hmC was downregulated in pancreatic tumors (Fig. 1d).

In previous studies, TET1 was reported to play an important role in regulating 5-hmC levels in solid tumors; therefore, we calculated the correlativity between TET1 and 5-hmC. As expected, both Kendall’s tau-b analysis and Spearman analysis showed TET1 was positively correlated with 5-hmC, while there were no statistically significant correlation with TET2 and TET3 (Table 1, Additional file 2: Figure S1a). Considering this result, we focused our subsequent research on the role of TET1 in pancreatic tumors.

**Table 1:** Correlation between 5hmC level and TET family levels

Sample#	5hmc	TET1		TET2		TET3	
	Rank	log (T/N,10)	Rank	log (T/N,10)	Rank	log (T/N,10)	Rank
1	1*	0.21	1	−0.21	− 1	− 0.4	− 1
2	− 1*	− 0.48	− 1	− 0.26	− 1	− 0.54	− 1
3	1	0.15	1	− 0.04	− 1	0.28	1
4	1	0.16	1	0.20	1	0.31	1
5	−1	−0.53	− 1	− 0.49	− 1	− 0.46	− 1
6	1	0.12	1	−0.12	− 1	− 0.02	− 1
7	− 1	− 0.52	− 1	0.24	1	0.1	1
8	1	0.39	1	0.12	1	−0.29	−1
9	− 1	−0.52	− 1	0.38	1	−0.19	− 1
10	1	0.07	1	−0.22	−1	−0.09	− 1
11	−1	−0.74	− 1	− 0.39	− 1	0.28	1
12	−1	−0.04	− 1	−0.02	− 1	− 0.14	−1
13	−1	− 0.33	− 1	− 0.12	−1	− 0.51	− 1
14	1	−0.56	−1	− 0.58	− 1	0.41	1
15	−1	−0.45	−1	0.27	1	−0.35	−1
Kendall’s tau-b							
coefficient			0.873		−0.094		0.189
P			0.001		0.724		0.480
Spearman							
coefficient		0.650		−0.139		0.449	
P		0.009		0.62		0.093	

*1 represents the levels of 5-hmC, TET1, TET2, and TET3 in tumor tissues that were higher in non-tumor tissues, −1 indicates the opposite

To analyze the correlation between TET1 expression levels and clinic-pathological parameters, we implemented a tissue array containing 63 pancreatic tumor tissues (57 of 63 had paired neighboring non-tumor tissues). IHC showed that TET1 was reduced in 35/57 pancreatic tumor tissues compared with adjacent non-tumor tissues, which was consistent with the mRNA level results (Fig. 1e). Survival analysis showed pancreatic cancer patients with low TET1 expression had shorter overall survival than patients with high TET1 levels (Fig. 1f). No significant correlation was found between TET1 expression and other clinical parameters (Table 2).

**Table 2:** Correlation between TET1 expression level and clinic-pathological parameters

Clinical-pathologic parameters		TET1 expression		*p* value
		Low (*N* = 32)	High (*N* = 31)	
Age (years)	≥60	24	18	*P* = 0.154
	<60	8	13	
Gender	male	18	18	*P* = 0.884
	female	14	13	
Tumor size (cm)	≥5 cm	10	8	*P* = 0.663
	<5 cm	22	23	
pathological grading	I,II	17	17	*P* = 0.891
	III,IV	15	14	
Nerve metastasis	Yes	14	13	P = 0.884
	No	18	18	
Lymph node metastasis	Yes	12	13	*P* = 0.719
	No	20	18	
Distant metastasis	Yes	2	2	*P* = 0.974
	No	30	31	
TNM stage*	IA,IB,IIA	20	17	*P* = 0.537
	IIB,III,IV	12	14	

*TNM stage was classified according to the AJCC Staging Manual (7th Edition)

### TET1 suppresses pancreatic cell proliferation in vitro and in vivo

**Fig. 2:**
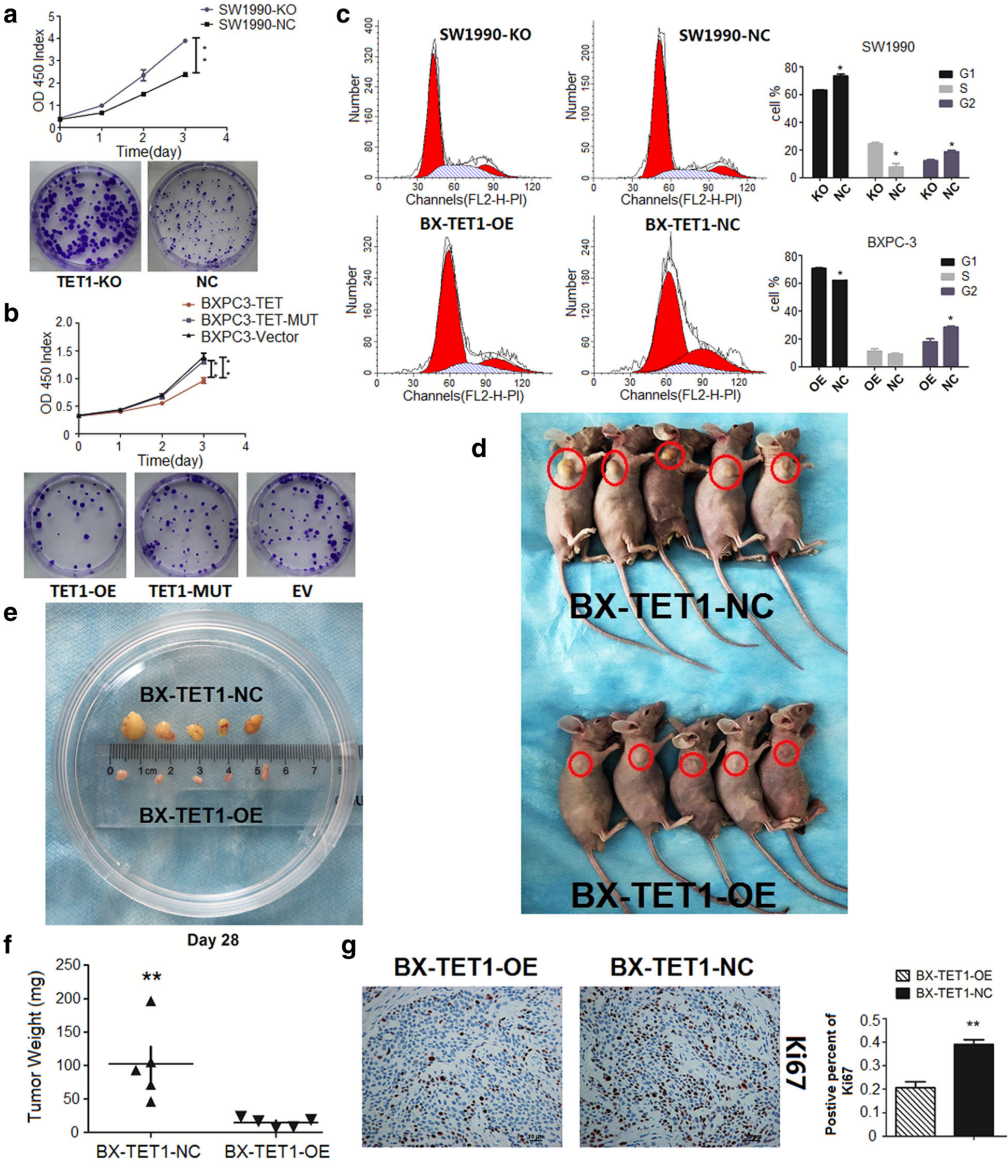
TET1 suppresses pancreatic cell proliferation in vitro and in vivo. **a** In SW1990-KO, TET1 knockout strongly accelerated cell proliferation (**P < 0.01, up CCK8, down colony formation assay). **b** TET1 overexpressed in BXPC-3 by TET1-OE transient transfection inhibited cell proliferation (*P* < 0.01, up CCK8, down colony formation assay) compared to TET1-MUT and EV transient transfection. **c** Flow cytometry analysis of the cell cycle distribution of SW1990-KO and BX-TET1-OE cells compared with their negative control cells, respectively (left). Data summary (*P < 0.05, right). **d** and **e** Subcutaneous tumor formation (red loops) of BX-TET1-OE and BX-TET1-NC cells. **f** Tumor weight comparison (**P < 0.01). **g** Ki-67 IHC analysis of tumors implanted in nude mice (mag. 200×, left). Data summary (**P < 0.01, right)

To investigate the function of TET1 in pancreatic tumors, we selected SW1990 and BXPC-3 pancreatic cell lines for further study (Additional file 2: Figure S1b). We utilized CRISPR/CAS9 to knock out TET1 in SW1990 cells to build a stable TET1 KO cell line (SW1990-KO), and built a stable TET1 overexpressing cell line using the dCAS9-SAM system in BXPC-3 cells (BX-TET1-OE). We confirmed the efficiency by western blotting, and further verified this by dot blotting (Additional file 2: Figure S1c, S1d). CCK8 and colony formation assays showed growth of SW1990 cells was significantly elevated when TET1 was knocked out (*P* < 0.01), revealing that TET1 strongly suppressed cell proliferation (Fig. 2a).

To determine the role of TET1 hydroxymethylation, we constructed a plasmid (TET1-mut) expressing a hydroxylase-deficient mutant TET1 (H1672Y/D1674A) matching the wild type TET1 overexpressing plasmid. TET1-mut transient transfection induced a significant elevation of TET1 but no change in levels of 5-hmC (Additional file 2: Figure S1e), as well as loss of suppression of cell proliferation (Fig. 2b). Overexpression of wild type TET1 strongly inhibited cell vitality (TET1-OE vs EV, *P* < 0.01), while there was no difference between TET1-mut overexpressing cells and EV transiently transfected cells (TET1-MUT vs EV, *P* > 0.05), indicating that stabilization of the catalytic domain was an essential anchor for TET1 suppression of cell growth.

Cell cycle analysis showed TET1-KO decreased the number of cells in G0/G1 phase by 10% (SW1990-KO 63% vs SW1990-NC 73%, *P* < 0.05), and TET1-OE increased the number of cells in G0/G1 phase by 8% (BX-TET1-OE 70% vs BX-TET1-NC 62%, *P* < 0.05), indicating TET1 can induce GO/G1 arrest, contributing to the suppression of the cell growth promotion of TET1 (Fig. 2c).

The in vitro proliferation assay exhibited the tumor-suppressive function of TET1. We then implanted BX-TET1-OE and BX-TET1-NC cells subcutaneously into nude mice to investigate the role of TET1 in tumor growth suppression in vivo. In accordance with the in vitro results, TET1 overexpression impaired tumor growth in the animal model; volumes and weights of implanted tumors were significantly decreased in the TET1 OE group compared to the NC group (*P* < 0.01) (Fig. 2d-f). Furthermore, IHC of implanted tumors showed that the number of Ki-67 positive cells also declined in the TET1 OE group as compared to the NC group (Fig. 2g). These results demonstrate that TET1 functioned as a tumor proliferation suppressor gene in pancreatic tumors.

### TET1 inhibits pancreatic cell migration, invasion, and EMT

**Fig. 3:**
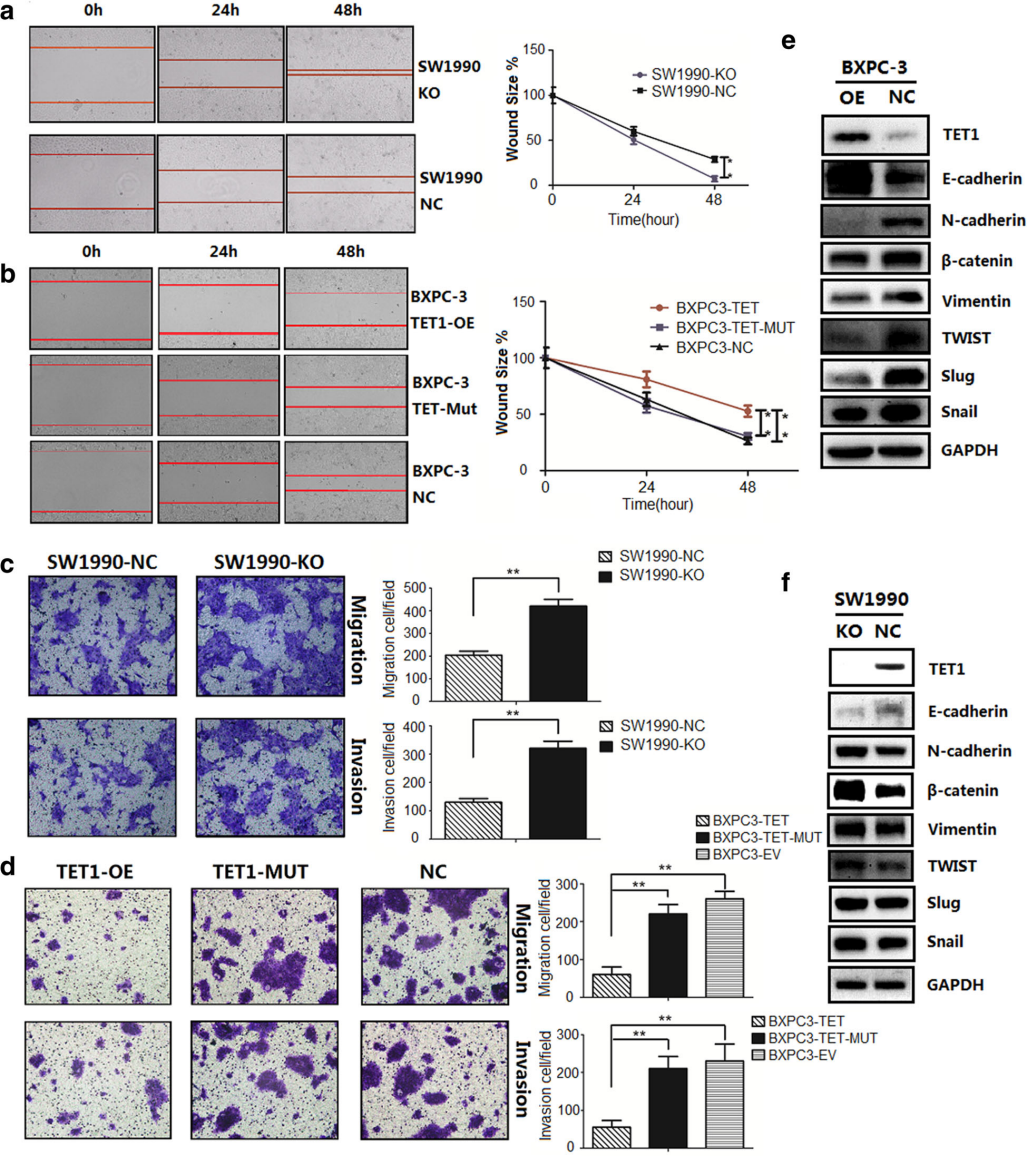
TET1 suppresses pancreatic cell migration, invasion, and EMT. **a** and **b** Wound healing assay for TET1-KO, TET1-OE, TET1-MUT, and NC cell migration at 0, 24, and 48 h (left), calculated data (right), **P < 0.01. **c** and **d** Transwell assays revealed that overexpression of wild type but not mutant TET1 inhibited cell migration and invasion, while TET1 knockout accelerated migration and invasion, ** *P* < 0.01. **e** and **f**. Western blot analysis of epithelial markers and mesenchymal markers in BXPC-3 and SW1990 cells

In addition to tumor proliferation, we also investigated the role of TET1 in pancreatic cell migration, invasion, and EMT through wound healing and transwell assays. In the former, wound healing was reduced in the TET1-OE group (*P* < 0.01) and accelerated in the TET1-KO group (*P* < 0.01) compared to their respective NC groups (Fig. 3a, b). Identical results were found in the transwell assay, in which TET1 overexpression weakened pancreatic cell migration and invasion through transwell membranes (*P* < 0.01), while TET1 catalytic domain mutation resulted in loss of this function (Fig. 3c, d). These results show that TET1 can inhibit migration and invasion in pancreatic tumors.

As EMT plays an important role in initiating tumor metastasis, to confirm whether TET1 suppresses migration and invasion by inhibiting EMT, we compared the levels of E-cadherin, an EMT marker, in cell lines by western blotting (Fig. 3e, f). Compared with the NC group, E-cadherin was upregulated and the mesenchymal markers N-cadherin, β-catenin, Vimentin, Twist, Slug, and Snail were downregulated in the TET1-OE group. Consistent results were found in the TET1-KO group, the E-cadherin was downregulated and the mesenchymal markers were upregulated. We also observed the morphology change of cells after TET1 knock-out: the SW1990-KO cells showed a long spindle shape with less cell-to-cell contact than SW1990-NC cells (Additional file 3: Figure S2a). The expression of these markers indicates that TET1 reverses EMT progress in pancreatic cancer.

### TET1 reverses EMT progress via the Wnt/β-catenin signaling pathway

**Fig. 4:**
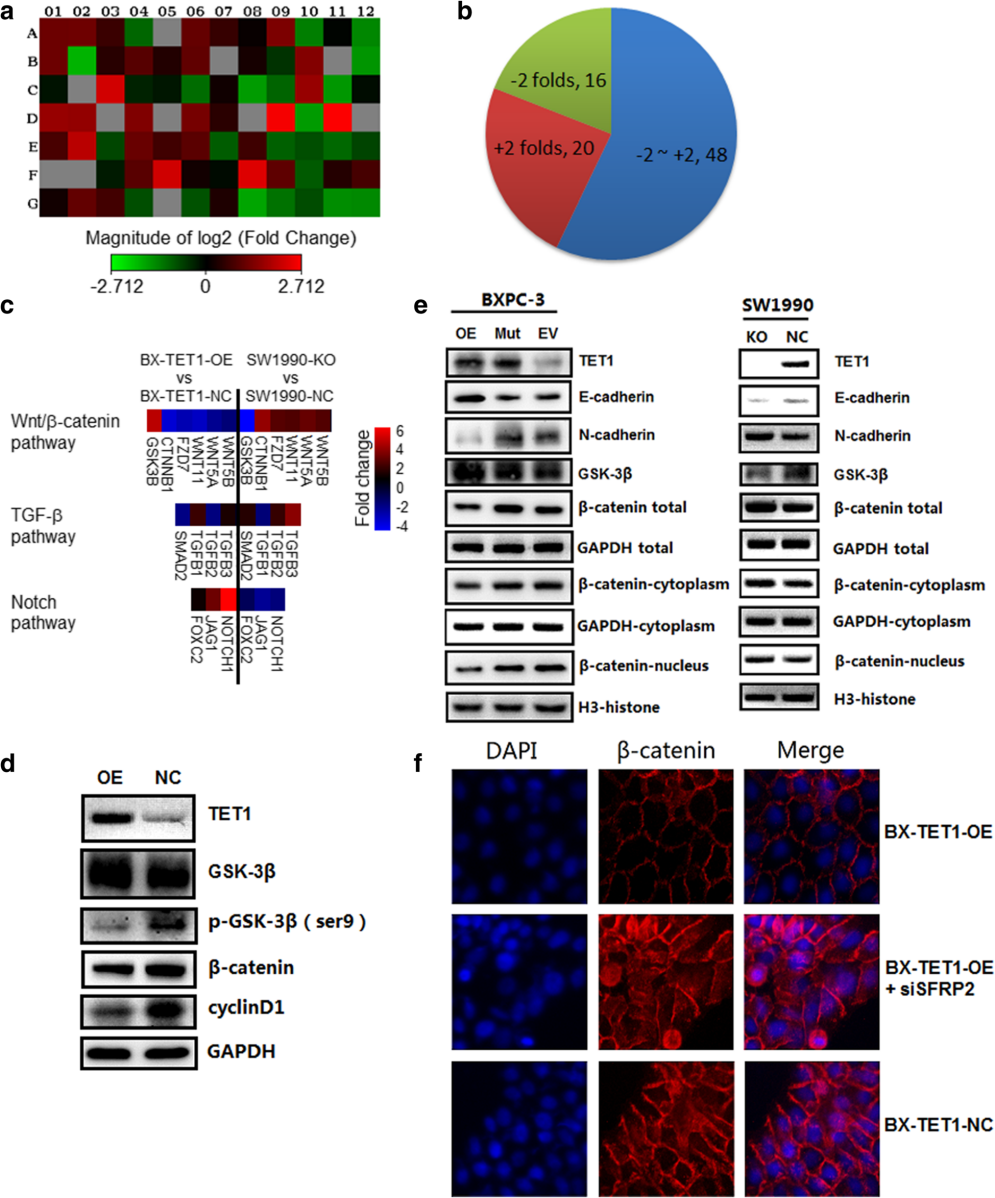
TET1 reverses EMT progress via the Wnt/β-catenin signaling pathway. **a** and **b** RNA array analysis of 84 EMT-associated genes in BX-TET1-OE and BX-TET1-NC cells, shown as a heatmap (log2-fold changes). **c** Heatmap (fold changes) of Wnt/β-catenin, TGF-β, and NOTCH signaling pathway changes in BX-TET-OE and SW1990-KO cells comparing respective negative controls. **d** Western blot analysis of key changes in gene expression in the Wnt/β-catenin pathway in BX-TET1-OE and BX-TET1-NC cells. **e** Western blot analysis of total β-catenin distribution in BXPC-3 and SW1990 cells. **f** Immunofluorescence staining of intracellular migration of active β-catenin in BX-TET1-OE and BX-TET1-NC cells

Several signaling pathways, including TGF-β, Wnt/β-catenin, Notch, FGF (Fibroblast growth factor), EGF (Epidermal growth factor), and HGF (Hepatocyte growth factor), as well as hypoxia can induce EMT in tumor progression. To identify the signaling pathways influencing EMT, we performed RNA array analysis of 84 EMT-associated genes in pancreatic cell lines (Fig. 4a, Tables 3 and 4, Additional file 3: Figure S2b). In the RNA array report of TET1 over expression, 20 EMT-associated genes were upregulated > 2-fold when TET1 was overexpressed, and 16 genes were downregulated by ~ 2-fold (Fig. 4b). Expression of β-catenin, Vimentin, Twist, Slug, Snail was in accordance with the results of western blot analysis, while occludin expression was elevated by 6-fold. In the TGF-β pathway, Smad2 and TGF-β2 were downregulated by ~ 2-fold, while TGF-β1 and TGF-β3 were upregulated by 2-fold and 1.30-fold, respectively, showing an unstable change in this pathway (Fig. 4c). In the Notch pathway, Jagged1 (JAG1) and NOTCH1 were upregulated by 3.07-fold and 6.17-fold, respectively, and Forkhead box C2 (FOXC2) was unaltered (Fig. 4c). In the Wnt/β-catenin pathway, Catenin beta-1 (CTNNB1), Frizzled family receptor 7 (FZD7), Wingless-type MMTV integration site family 11 (WNT11), WNT5A, and WNT5B were downregulated by 3.75-fold, 3.03-fold, 3.40-fold, 2.32-fold, and 1.75 fold, respectively, while Glycogen synthase kinase 3 beta (GSK-3B) was upregulated, revealing a strong inhibition (Fig. 4c). Meanwhile the RNA array report of TET1 knock-out revealed the Wnt/β-catenin pathway was activated, the Notch pathway was suppressed and the TGF-β pathway also showed an unstable change (Fig. 4c, Table 4). As the Notch signaling pathway is an activator of EMT, we therefore focused on inhibition of the Wnt/β-catenin pathway.

**Table 3:** RNA array analysis of 84 EMT-associated genes in BX-TET1-OE and NC cells*

	1	2	3	4	5	6	7	8	9	10	11	12
A	AHNAK	AKT1	BMP1	BMP2	BMP7	CALD1	CAMK2N1	CAV2	CDH1	CDH2	COL1A2	COL3A1
	2.22	2.23	1.53	−2.13	1.04	2.08	1.5	1.07	2.49	−2.53	−1.03	−2.72
B	COL5A2	CTNNB1	DSC2	DSP	EGFR	ERBB3	ESR1	F11R	FGFBP1	FN1	FOXC2	FZD7
	2.23	−3.75	1.34	1.88	1.29	2.02	1.04	1.18	−1.45	2.64	1.04	−3.03
C	GNG11	GSC	GSK3B	IGFBP4	IL1RN	ILK	ITGA5	ITGAV	ITGB1	JAG1	KRT14	KRT19
	−1.14	1.04	4.7	−1.28	−1.18	−2.22	1.13	−3.17	− 2.1	3.07	− 3.07	− 1.09
D	KRT7	MAP 1B	MMP2	MMP3	MMP9	MSN	MST1R	NODAL	NOTCH1	NUDT13	OCLN	PDGFRB
	3.07	2.97	1.04	2.58	1.04	2.4	1.37	1.04	6.17	−3.18	6.55	1.04
E	PLEK2	DESI1	PTK2	PTP4A1	RAC1	RGS2	SERPINE1	GEMIN2	SMAD2	SNAI1	SNAI2	SNAI3
	1.7	3.96	−1.67	2.16	1.61	1.94	−1.96	1.57	−2.03	−1.76	−2.16	−1.65
F	SOX10	SPARC	SPP1	STAT3	STEAP1	TCF3	TCF4	TFPI2	TGFB1	TGFB2	TGFB3	TIMP1
	1.04	1.04	−1.21	1.91	4.6	1.14	−1.08	5.12	2	−1.93	1.3	1.66
G	TMEFF1	TMEM132	TSPAN13	TWIST1	VCAN	VIM	VPS13A	WNT11	WNT5A	WNT5B	ZEB1	ZEB2
	1.14	2.06	1.62	−1.77	1.04	−1.82	1.24	−3.4	−2.32	−1.75	−2.85	−2.76

*RNA array analysis of 84 EMT-associated genes in BX-TET1-OE and NC cells, shown as a table (fold changes)

**Table 4:** RNA array analysis of 84 EMT-associated genes in SW1990-KO and NC cells*

	1	2	3	4	5	6	7	8	9	10	11	12
A	AHNAK	AKT1	BMP1	BMP2	BMP7	CALD1	CAMK2N1	CAV2	CDH1	CDH2	COL1A2	COL3A1
	−1.55	−1.90	−1.57	1.31	1.03	−1.27	−1.54	1.01	−2.49	2.66	1.03	1.01
B	COL5A2	CTNNB1	DSC2	DSP	EGFR	ERBB3	ESR1	F11R	FGFBP1	FN1	FOXC2	FZD7
	−2.27	3.90	−1.60	−1.38	−1.10	1.30	1.03	−1.01	1.78	−1.96	−1.13	2.58
C	GNG11	GSC	GSK3B	IGFBP4	IL1RN	ILK	ITGA5	ITGAV	ITGB1	JAG1	KRT14	KRT19
	1.34	1.03	−4.67	1.17	1.03	1.22	1.65	1.78	1.80	−2.41	1.03	3.11
D	KRT7	MAP 1B	MMP2	MMP3	MMP9	MSN	MST1R	NODAL	NOTCH1	NUDT13	OCLN	PDGFRB
	−1.49	−1.64	1.80	2.38	3.53	−1.82	−1.28	1.03	−1.83	1.95	−2.57	−3.18
E	PLEK2	DESI1	PTK2	PTP4A1	RAC1	RGS2	SERPINE1	GEMIN2	SMAD2	SNAI1	SNAI2	SNAI3
	−1.41	−2.04	2.76	−2.27	−1.35	− 1.61	2.11	− 1.30	1.55	1.76	1.41	1.62
F	SOX10	SPARC	SPP1	STAT3	STEAP1	TCF3	TCF4	TFPI2	TGFB1	TGFB2	TGFB3	TIMP1
	1.03	1.18	1.03	−1.17	1.03	1.83	2.99	1.94	−1.76	1.88	3.68	−1.34
G	TMEFF1	TMEM132	TSPAN13	TWIST1	VCAN	VIM	VPS13A	WNT11	WNT5A	WNT5B	ZEB1	ZEB2
	−1.08	−1.20	−1.23	2.14	1.03	2.87	1.50	2.42	2.93	2.50	1.44	1.55

*RNA array analysis of 84 EMT-associated genes in SW1990-KO and SW1990-NC cells, shown as a table (fold changes)

To investigate the molecular function underlying the inhibition of the Wnt/β-catenin pathway by TET1, we determined the levels of GSK-3β and β-catenin corresponding to different levels of TET1. We found overexpression of TET1 increased total GSK-3β, while phospho-GSK-3β (Ser9) was decreased (Fig. 4d). Cyclin D1, a protein downstream β-catenin, was also downregulated when TET1 was overexpressed (Fig. 4d). As the total β-catenin levels were detected, we separated nuclear protein and cytosol protein fractions to determine intracellular migration of β-catenin. Western blot analysis showed that β-catenin was decreased in both the nucleus and cytoplasm in TET1-overexpressing cells, and was increased in both the nucleus and cytoplasm in TET1 knock-out cells (Fig. 4e), indicating that TET1 downregulated β-catenin, and inhibited β-catenin migration to the nucleus. To verify this mechanism, immunofluorescence staining was repeated, and revealed the intracellular migration of active β-catenin as expected (Fig. 4f).

Taken together, these results demonstrate that TET1 suppresses EMT progression via the Wnt/β-catenin signaling pathway.

### TET1-dependent demethylation induces transcriptional activation of SFRP2

**Fig. 5:**
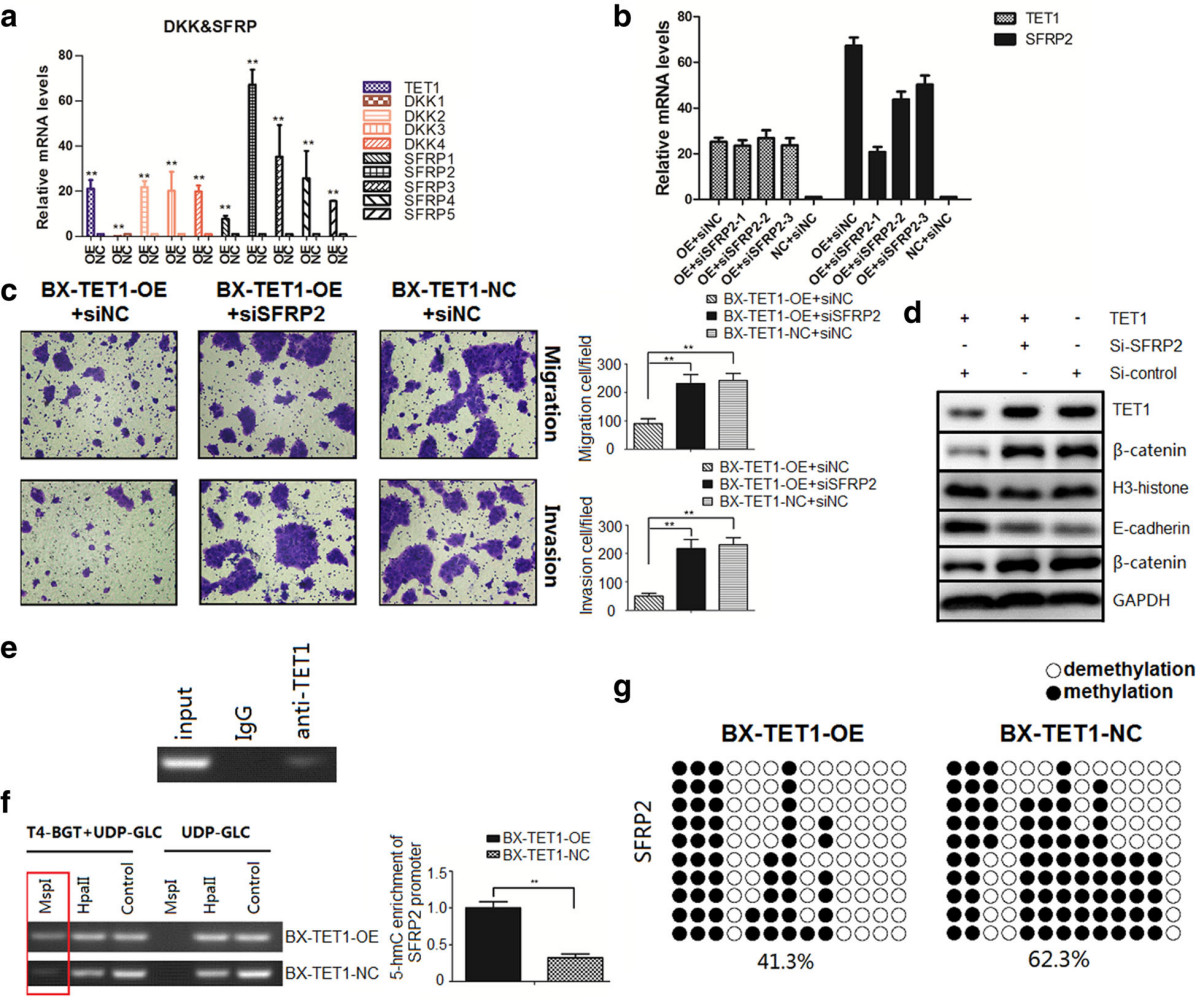
TET1-dependent demethylation induces transcriptional activation of SFRP2. **a** QRT-PCR of mRNA levels of DKKs and SFRPs in BX-TET1-OE and BX-TET1-NC cells. **b** Efficiency of si-SFRP2 was detected by qRT-PCR. **c** Transwell assays revealed that SFRP2 knockdown diminished TET1 inhibition of cell migration and invasion, **P < 0.01. **d** Western blot analysis of total β-catenin distribution after SFRP2 knockdown in BX-TET1-OE and BX-TET1-NC cells. **e** ChIP analysis of TET1 binding to the SFRP2 promoter. **f** 5-hmC and 5-mC content analysis in the SFRP2 promoter; *Msp*I digestion indicated the 5-hmC level, *Hpa*II digestion indicated 5-hmC and 5-mC levels, and the control indicated the total amount of 5-C, **P < 0.01. **g** BSP indicated the changes in methylation levels in the SFRP2 promoter

RNA array analysis revealed that Frizzled 7 (FZD7) was downregulated when TET1 was overexpressed. As a receptor of the Wnt/β-catenin signal, FZD7 has been shown to be highly expressed in a variety of cancers, including gastric cancer and breast cancer [22, 23], and is involved in regulating cancer cell proliferation, migration, and invasion via the activating Wnt/β-catenin pathway. We determined if TET1 induces transcriptional activation of Frizzled inhibitors by comparing the mRNA levels of Dickkopf-related proteins (DKKs) and secreted Frizzled-related proteins (SFRPs) between BXPC3-TET1-OE and NC cells. With the exception of DKK1, which was silenced, DKK2, DKK3, DKK4 and SFRPs were significantly upregulated, especially SFRP2, which was enhanced by 67-fold (Fig. 5a). Therefore, we decided to focus on the correlation between SFRP2 and TET1.

To investigate the role of SFRP2 in TET1-dependent inhibition of the Wnt/β-catenin pathway, we knocked down SFRP2 in TET1 overexpressing cells by siRNAs, and selected the most efficient siSFRP2–1 for subsequent experiments (Fig. 5b). We found the TET1-dependent reversion of EMT was diminished when SFRP2 was knocked down (Fig. 5c). Subsequently, nuclear β-catenin was recovered when SFRP2 was knocked down (Figs. 4f, 5d). Immunofluorescence staining correspondingly revealed active β-catenin. The classical molecular mechanism underlying the upregulation of target genes by TET1 consists of TET1 binding to the promoter of the target gene, catalyzing 5-mC hydroxylation to 5-hmC in CpG islands, inducing demethylation, and ultimately activating gene transcription. In accordance with this mechanism, we first performed ChIP analysis and found TET1 directly bound the SFRP2 promoter (Fig. 5e). Then, we analyzed 5-hmC and 5-mC enrichment in the SFRP2 promoter, which revealed that 5-hmC levels were significantly increased by TET1 overexpression (Fig. 5f). Bisulfate sequencing PCR (BSP) also exhibited consistent results (Fig. 5g).

Together, these results demonstrate that TET1 directly binds to the SFRP2 promoter, catalyzing demethylation via conversion of 5-mC to 5-hmC, inducing transcriptional activation of SFRP2, and ultimately inhibiting the Wnt/β-catenin signaling pathway.

### TET1 suppresses pancreatic tumor metastasis in vivo

**Fig 6:**
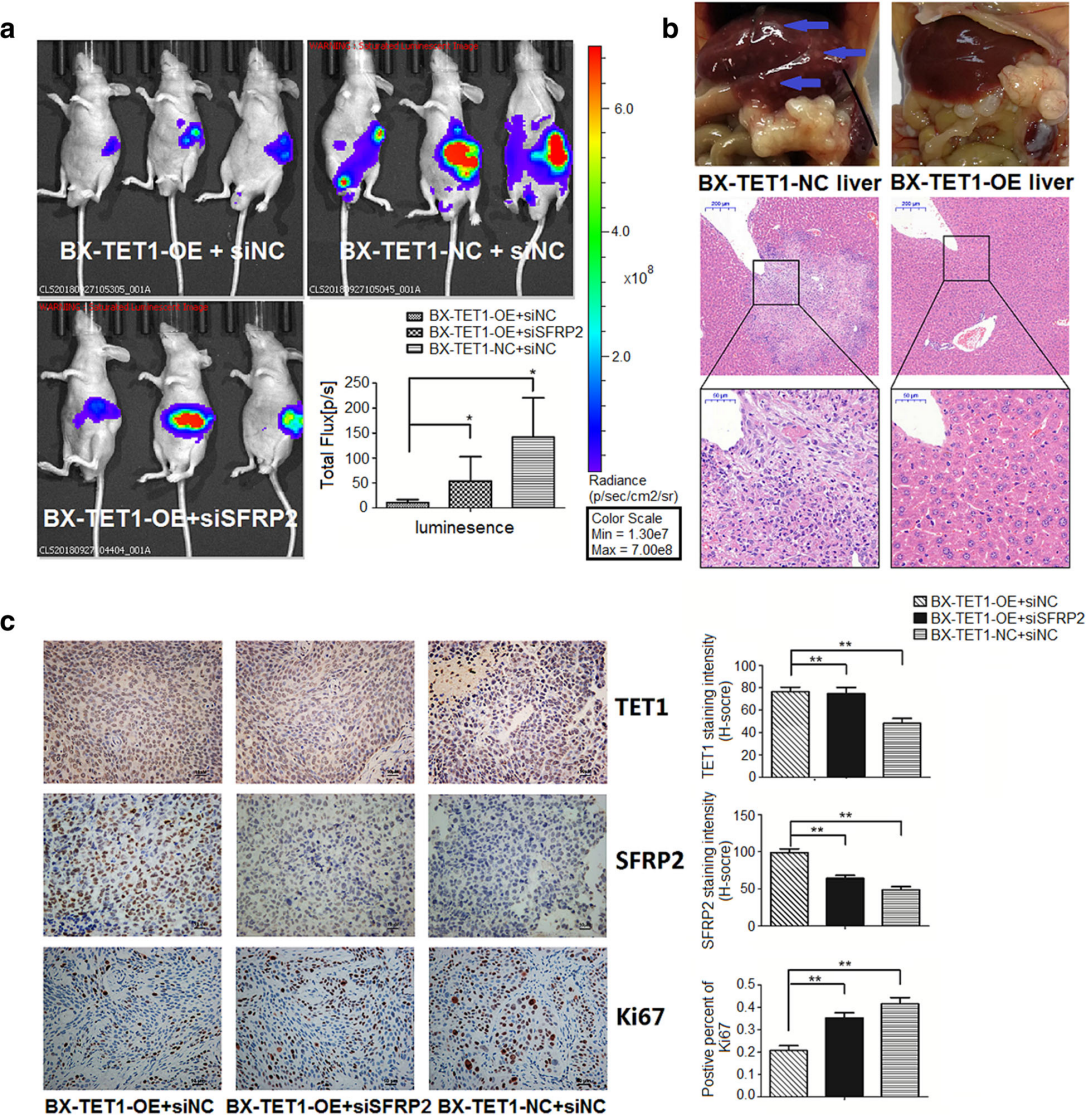
TET1 suppresses pancreatic tumor metastasis in vivo. **a** Metastasis in the orthotopic pancreatic metastatic model in nude mice was measured by bioluminescent signal on a bioluminescence IVIS. *P < 0.05. **b** Liver photos and H&E staining revealed distant metastasis in liver (blue arrows) (Original magnification, 20×. Enlarged graph, 80×). **c** IHC analysis of orthotopic implanted pancreatic tumors in nude mice (mag. 200×, left). Data summary (**P < 0.01, right)

To simulate pancreatic tumor metastasis, we established an orthotopic pancreatic metastatic model in nude mice. BX-TET1-OE and BX-TET1-NC were labeled with firefly luciferase and injected into the pancreata of nude mice, and an additional group were injected with si-SFRP2 transfected BX-TET1-OE cells. An additional abdominal injection of 200 nmol/kg si-SFRP2 was performed after 1 week, while 200 nmol/kg si-NC were injected into other two groups. After 3 weeks, metastasis was determined via the bioluminescent signal detected by a bioluminescence IVIS (Fig. 6a). The BX-TET1-OE signal was significantly diminished compared to the control group, and the si-SFRP2 group signal was enhanced compared to the BX-TET1-OE group. Furthermore, H&E staining revealed liver metastasis were suppressed in the BX-TET1-OE group compared to the BX-TET1-NC group (Fig. 6b). The percent of Ki-67 positive cells was significantly higher in tumors in the si-SFRP2 group compared to that in the BX-TET1-OE group (Fig. 6c). This in vivo result further verifies the role of TET1 in inhibiting the Wnt/β-catenin signaling pathway.

### TET1 increases pancreatic tumor suppressor genes in pancreatic cell lines

As TET1 is a demethylation enzyme, we hypothesized that pancreatic tumor suppressor genes could be involved in the anti-tumor activity of TET1. The tumor suppressors p16, Ras association domain-containing protein 1 (RASSF1A), and preproenkephalin (ppENK) have been correlated to abnormal hypermethylation in pancreatic tumor [24, 25]. We detected the mRNA levels of p16, RASSF1A, and ppENK in pancreatic cells, and found only p16 was upregulated in response to TET1 overexpression (Additional file 3: Figure S2c). Western blotting revealed that p16 was also elevated at the protein level in TET1 overexpressing cells (Additional file 3: Figure S2d). p16 has been reported to regulate cell cycle progression by inhibiting cyclin D-CDK4(Cyclin-dependent kinase 4) and cyclin D-CDK6 complexes responsible for initiating G1/S phase transition. In a previous study, we found that TET1 also arrests the cell cycle in the G0/G1 phase; thus, upregulation of p16 may be involved in this phenomenon. However, the underlying mechanism needs to be further investigated.

## Discussion

As the major form of DNA epigenetic modification, 5-mC is modified in > 60% CpG sites in the mammalian genome, and plays an important role in differentiation and disease [26]. Members of the TET family convert 5-mC to 5-hmC, initiating DNA demethylation, and ultimately generating 5-C with the help of thymine DNA glycosylase (TDG) and base excision repair (BER) [3]. Recently, TET-mediated DNA demethylation was widely studied in pluripotency, differentiation, and cancers [6–8, 13, 14]. In this study, we first demonstrated the involvement of TET1 in pancreatic tumors. We found TET1 was downregulated in pancreatic tumor tissues and cell lines, and that pancreatic tumor patients with low TET1 levels have shorter overall survival than patients with high levels of TET1. TET1 suppressed pancreatic tumor proliferation, migration, and invasion in vivo and in vitro via inhibiting the Wnt/β-catenin signaling pathway, which is consistent with results found for colon and ovarian cancer [27, 28].

EMT can be induced by various signaling pathways (TGF-β, Wnt/β-catenin, Notch, FGF, EGF, and HGF) and hypoxia in tumor progression [29]. Here, we performed RNA array analysis to determine the relevant EMT signaling pathway, specifically identifying the Wnt/β-catenin pathway. Although mRNA expression of JAG1 and NOTCH1 in the NOTCH pathway were upregulated in the TET1 overexpressing group, the NOTCH pathway is considered to be an activator of EMT [29], and cross-talk between the NOTCH and Wnt/β-catenin pathways did not support the phenotype in our study; thus, we focused on the Wnt/β-catenin pathway. In pancreatic tumors, we revealed that TET1 diminished β-catenin in both the nucleus and cytoplasm and disturbed the intracellular migration of active β-catenin. Levels of cyclin D1, a downstream target of β-catenin, were also decreased. These results provide strong evidence that TET1 suppresses EMT in pancreatic tumors by inhibiting Wnt/β-catenin.

Wnts bind to FZD receptors and lipoprotein receptor-related protein 5/6 (LRP5/6) co-receptors to form a complex that initiates canonical Wnt signaling (β-catenin-dependent signaling). The mRNA levels of Wnt pathway-associated genes, including *CTNNB1*, *FZD7*, *WNT11*, *WNT5A*, and *WNT5B*, were found to be downregulated, and *GSK-3B* was upregulated. *CTNNB1* encodes β-catenin, *GSK-3B* encodes GSK-3β, and both are key in the Wnt/β-catenin pathway. The *FZD7* gene encodes Frizzled-7, a member of the Frizzled family, is a receptor of Wnt signaling [30], and has been reported to be an oncogene in various cancers [22, 23]. *WNT11*, *WNT5A*, and *WNT5B* belong to the non-canonical Wnt signaling pathway (β-catenin-independent signaling), and are involved in oncogenesis and in several developmental processes [31–33]. Expression of these oncogenes were consistently suppressed in our study, verifying the inhibition of both canonical and non-canonical Wnt signaling. As antagonists of the Wnt pathway, DKKs and SFRPs competitively bind the Wnt-binding site of Frizzled proteins to modulate Wnt signaling [34]. In our study, DKKs and SFRPs were all upregulated in response to TET1 overexpression except DKK1, which is a similar result obtain by two other studies in colon and ovarian cancer [27, 28]. The level of SFRP2 mRNA was elevated by ~ 70-fold. Following the classical molecular mechanism of TET1-mediated transcriptional activation, we demonstrated that TET1 binds directly to the SFRP2 promoter, catalyzes 5-mC hydroxylation to 5-hmC in the promoter CpG islands, induces demethylation, initiates SFRP2 transcriptional activation, and ultimately inhibits EMT in pancreatic tumors. Except TET1, other molecular mechanisms inhibiting Wnt signaling in pancreatic tumor like FOXO1-related LINC01197 has been reported [35], these mechanisms may contributes the final activity of Wnt signaling pathway in pancreatic tumor.

In our study, the inhibitory function of TET1 in pancreatic tumor proliferation was detected in vivo and in vitro. Arrest of the cell cycle in G0/G1 phase indicates the cell cycle-associated proteins cyclin D1 and p16 may be involved in this mechanism; however, this this needs to be further investigated. As anti-oncogenes, p16, RASSF1A, and ppENK have been associated with aberrant hypermethylation in pancreatic tumors [24, 25]. Previous studies have shown that p16 methylation is correlated with an increased risk of pancreatic cancer [36], and DNA hypermethylation of the promoter was observed in 60% of p16 genes, and was markedly correlated with decreased mRNA expression [37]. The ppENK gene methylation level was found to be increased by < 90% in pancreatic cancer [24]; methylated ppENK was detected in all pancreatic cancer cell lines tested, and was associated with loss of mRNA expression in pancreatic carcinoma cell lines and normal pancreatic tissues [38]. RASSF1A hypermethylation was detected in 29 out of 45 (64%) primary adenocarcinomas, 10 out of 12 (83%) endocrine tumors, and 8 out of 18 (44%) pancreatitis samples [25]. Therefore, we investigated expression of these genes, and found the expression of ppENK and RASSF1A were not influenced by TET1, only p16 was upregulated at the mRNA and protein levels. As an inhibitor of cyclin-dependent kinases (CDK), p16 slows down the cell cycle by prohibiting progression from G1 phase to S phase [39]. The upregulation of p16 may have contributed to the G0/G1 arrest observed in our study; however, the underling mechanism remains to be elucidated.

Targeted therapy is a therapeutic strategy with more effective treatments and reduced toxicity, but the limited knowledge of potential targets suppresses its application scope in tumor patients [40]. For therapy of pancreatic tumor patients with TET1 low expressing, our finds provide potential targets including TET1 and its downstream gene SFRP2 for therapy. CRISPR–Cas9-based genome editing provides a new therapeutic strategy with high specificity [41], such as the dCas9-multiGCN4/scFv-TET1CD-sgRNA-based SFRP2-targeted demethylation system provides a SFRP2-targeting therapeutic strategy to inhibit pancreatic tumor metastasis [42]. With the development of technology, more novel targeted therapeutic strategy will be available.

## Conclusion

TET1 is downregulated in pancreatic tumor tissues, and pancreatic tumor patients with low TET1 levels have shorter overall survival than patients with high levels of TET1. TET1 suppresses pancreatic tumor proliferation and metastasis. TET1 binds to the SFRP2 promoter and catalyzes demethylation to activate SFRP2 transcription, inhibiting the Wnt signaling pathway and ultimately obstructing EMT in pancreatic tumors.

## Additional files

Additional file 1: **Table S1.** Primers in this study (DOCX 18 kb) (DOCX 19 kb)

Additional file 2: **Figure S1.** Correlation between TET1 and 5-hmC& construction of TET1 knockout/overexpression. **a** Correlation between TET1 and 5-hmC, shown as a heatmap (Kendall’s tau-b rank) **b** TET1 levels were determined in pancreatic cell lines by qRT-PCR and western blot. **c–e** Construction of cell lines are described in the Materials and Methods. Efficiency of wild type TET1 knockout and overexpression, and mutant TET1 overexpression was determined by Western blotting. 5-hmc content was analyzed by dot blotting. (BMP 1922 kb)

Additional file 3 : **Figure S2** EMT changes in cells with TET1 knock-out & Pancreatic tumor suppressor genes in pancreatic cell lines. **a** morphology changes after TET1 knock-out in SW1990. **b** RNA array analysis of 84 EMT-associated genes in SW1990-KO and SW1990-NC cells, shown as a heatmap (log2-fold changes). **c** mRNA levels of p16, RASSF1A, and ppENK in BX-TET1-OE cells compared to NC cells, as detected by qRT-PCR. **d** Western blot of p16 in BX-TET1-OE cells compared to NC cells. (BMP 1552 kb)

## Data Availability

All of the data and material in this paper are available when requested.

## Abbreviations

5-C: 5-cytosine
5-hmC: 5-hydroxymethylcytosine
5-mC: 5-methylcytosine
AHNAK: AHNAK nucleoprotein
AKT1: V-akt murine thymoma viral oncogene homolog 1
AML: Acute myeloid leukemia
BER: Base excision repair
BMP1: Bone morphogenetic protein 1
BMP2: Bone morphogenetic protein 2
BMP7: Bone morphogenetic protein 7
CALD1: Caldesmon 1
CAMK2N1: Calcium/calmodulin-dependent protein kinase II inhibitor 1
CAV2: Caveolin 2
CDH1: Cadherin 1, type 1, E-cadherin (epithelial)
CDH2: Cadherin 2, type 1, N-cadherin (neuronal)
CDK4: Cyclin-dependent kinase 4
COL1A2: Collagen, type I, alpha 2
COL3A1: Collagen, type III, alpha 1
COL5A2: Collagen, type V, alpha 2
CTNNB1: Catenin beta-1
CTNNB1: Catenin (cadherin-associated protein), beta 1, 88kDa
DESI1: PPPDE peptidase domain containing 2
DKK: Dickkopf-related protein
DNMT: DNA methyltransferase
DSC2: Desmocollin 2
DSP: Desmoplakin
EGFR: Epidermal growth factor receptor
EMT: Epithelial-mesenchymal transition
ERBB3: V-erb-b2 erythroblastic leukemia viral oncogene homolog 3 (avian)
ESR1: Estrogen receptor 1
F11R: F11 receptor
FGF: Fibroblast growth factor
FGFBP1: Fibroblast growth factor binding protein 1
FN1: Fibronectin 1
FOXC2: Forkhead box C2
FOXC2: Forkhead box C2 (MFH-1, mesenchyme forkhead 1)
FZD7: Frizzled family receptor 7
FZD7: Frizzled family receptor 7
GEMIN2: Survival of motor neuron protein interacting protein 1
GNG11: Guanine nucleotide binding protein (G protein), gamma 11
GSC: Goosecoid homeobox
GSK3B: Glycogen synthase kinase 3 beta
GSK-3B: Glycogen synthase kinase 3 beta
HGF: Hepatocyte growth factor
IGFBP4: Insulin-like growth factor binding protein 4
IL1RN: Interleukin 1 receptor antagonist
ILK: Integrin-linked kinase
ITGA5: Integrin, alpha 5 (fibronectin receptor, alpha polypeptide)
ITGAV: Integrin, alpha V (vitronectin receptor, alpha polypeptide, antigen CD51)
ITGB1: Integrin, beta 1 (fibronectin receptor, beta polypeptide, antigen CD29 includes MDF2, MSK12)
JAG1: Jagged1
JAG1: Jagged 1
KRT14: Keratin 14
KRT19: Keratin 19
KRT7: Keratin 7
MAP1B: Microtubule-associated protein 1B
MMP2: Matrix metallopeptidase 2 (gelatinase A, 72kDa gelatinase, 72kDa type IV collagenase)
MMP3: Matrix metallopeptidase 3 (stromelysin 1, progelatinase)
MMP9: Matrix metallopeptidase 9 (gelatinase B, 92kDa gelatinase, 92kDa type IV collagenase)
MSN: Moesin
MST1R: Macrophage stimulating 1 receptor (c-met-related tyrosine kinase)
NODAL: Nodal homolog (mouse)
NOTCH1: Notch 1
NUDT13: Nudix (nucleoside diphosphate linked moiety X)-type motif 13
OCLN: Occludin
PDGFRB: Platelet-derived growth factor receptor, beta polypeptide
PLEK2: Pleckstrin 2
ppENK: preproenkephalin
PTEN: phosphatase and tensin homolog
PTK2: PTK2 protein tyrosine kinase 2
PTP4A1: Protein tyrosine phosphatase type IVA, member 1
RAC1: Ras-related C3 botulinum toxin substrate 1 (rho family, small GTP binding protein Rac1)
RASSF1A: Ras association domain-containing protein 1
RGS2: Regulator of G-protein signaling 2, 24kDa
SERPINE1: Serpin peptidase inhibitor, clade E (nexin, plasminogen activator inhibitor type 1), member 1
SFRP2: Secreted frizzled-related protein 2
SMAD2: SMAD family member 2
SNAI1: Snail homolog 1 (Drosophila)
SNAI2: Snail homolog 2 (Drosophila)
SNAI3: Snail homolog 3 (Drosophila)
SOX10: SRY (sex determining region Y)-box 10
SPARC: Secreted protein, acidic, cysteine-rich (osteonectin)
SPP1: Secreted phosphoprotein 1
STAT3: Signal transducer and activator of transcription 3 (acute-phase response factor)
STEAP1: Six transmembrane epithelial antigen of the prostate 1
TCF3: Transcription factor 3 (E2A immunoglobulin enhancer binding factors E12/E47)
TCF4: Transcription factor 4
TDG: Thymine DNA glycosylase
TET: Ten-eleven translocation
TFPI2: Tissue factor pathway inhibitor 2
TGFB1: Transforming growth factor, beta 1
TGFB2: Transforming growth factor, beta 2
TGFB3: Transforming growth factor, beta 3
TGFβ: Transforming growth factor beta
TIMP1: TIMP metallopeptidase inhibitor 1
TMEFF1: Transmembrane protein with EGF-like and two follistatin-like domains 1
TMEM132A: Transmembrane protein 132A
TSPAN13: Tetraspanin 13
TWIST1: Twist homolog 1 (Drosophila)
VCAN: Versican
VIM: Vimentin
VPS13A: Vacuolar protein sorting 13 homolog A (S. cerevisiae)
WNT11: Wingless-type MMTV integration site family 11
WNT11: Wingless-type MMTV integration site family, member 11
WNT5A: Wingless-type MMTV integration site family, member 5A
WNT5B: Wingless-type MMTV integration site family, member 5B
ZEB1: Zinc finger E-box binding homeobox 1
ZEB2: Zinc finger E-box binding homeobox 2
