# Supplementary material for: TET1-mediated DNA hydroxymethylation activates inhibitors of the Wnt/β-catenin signaling pathway to suppress EMT in pancreatic tumor cells
Source: J Exp Clin Cancer Res. 2019 Aug 9;38:348. doi: 10.1186/s13046-019-1334-5 (PMC6688318; doi:10.1186/s13046-019-1334-5)
Supplement: Supplementary file 1 — Table S1. Primers in this study (DOCX 18 kb) (DOCX 19 kb) [file 13046_2019_1334_MOESM1_ESM.docx]

**Additional file 1: Table S1. Primers in this study**

| Primer | Sequence (5′-3′) | |
| --- | --- | --- |
| TET1 | F | TCATGGGTGTCCAATTGCTA |
|  | R | GATGAGCACCACCATCACAG |
| TET2 | F | TCAGCATCATCAGCATCACA |
|  | R | ACTCACCCATCGCATACCTC |
| TET3 | F | GGTGGGTGTGTCCAGGTACT |
|  | R | CCTGCTTCCATTCAGAGGAG |
| GAPDH | F | CTGACTTCAACAGCGACACC |
|  | R | TGCTGTAGCCAAATTCGTTG |
| RASSF1A | F | TGGGGAGGTGAACTGGGAC |
|  | R | ACACGGCACGCACTTGG |
| ppENK | F | GCGGTTCCTGACACTTTGC |
|  | R | GGGTGCTGGTGCCATCTT |
| CDKN2A | F | CACATTCGCTAAGTGCTCGG |
|  | R | TCTTTCTTCCTCCGGTGCTG |
| SFRP1 | F | GCTCAACAAGAACTGCCAC |
|  | R | CTTGTCACACTTAAGCATCTCG |
| SFRP2 | F | CATCGAATACCAGAACATGCG |
|  | R | GATGGTCTCGTCTAGGTCATC |
| SFRP3 | F | CTCTTGGTGGAAGGCTCTATAG |
|  | R | TTCTGACTCTGAGTGGAATCAC |
| SFRP4 | F | ATGTTCCTCTCCATCCTAGTGG |
|  | R | CTCCTCGTACTGCTCGATG |
| SFRP5 | F | CCAGTGACTTTGTGGTCAAAAT |
|  | R | CTTCTTTTTCTGGGCTCCAATC |
| DKK1 | F | TACCAGACCATTGACAACTACC |
|  | R | TCCATTTTTGCAGTAATTCCCG |
| DKK2 | F | ACCACACACTAAGATGTCACAT |
|  | R | AAAATTTCCAGCCCATGAGAAC |
| DKK3 | F | AGCTATCACAATGAGACCAACA |
|  | R | CATTTGTCCAGTCTGGTTGTTG |
| DKK4 | F | GAGGGAGAAAGTTGTCTGAGAA |
|  | R | ACTGGCTTACAAATTTTCGTCC |
