# Supplementary figures and images for: TET1-mediated DNA hydroxymethylation activates inhibitors of the Wnt/β-catenin signaling pathway to suppress EMT in pancreatic tumor cells
Source: J Exp Clin Cancer Res. 2019 Aug 9;38:348. doi: 10.1186/s13046-019-1334-5 (PMC6688318; doi:10.1186/s13046-019-1334-5)

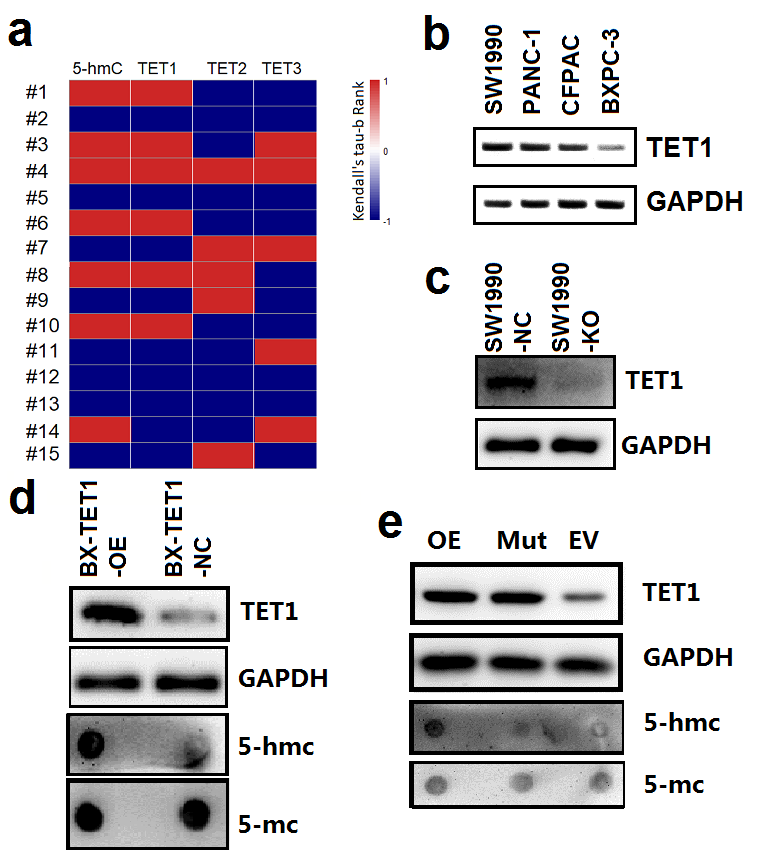

Supplement: Supplementary file 2 — Figure S1. Correlation between TET1 and 5-hmC& construction of TET1 knockout/overexpression. a Correlation between TET1 and 5-hmC, shown as a heatmap (Kendall’s tau-b rank) b TET1 levels were determined in pancreatic cell lines by qRT-PCR and western blot. c–e Construction of cell lines are described in the Materials and Methods. Efficiency of wild type TET1 knockout and overexpression, and mutant TET1 overexpression was determined by Western blotting. 5-hmc content was analyzed by dot blotting. (BMP 1922 kb) [file 13046_2019_1334_MOESM2_ESM.bmp]

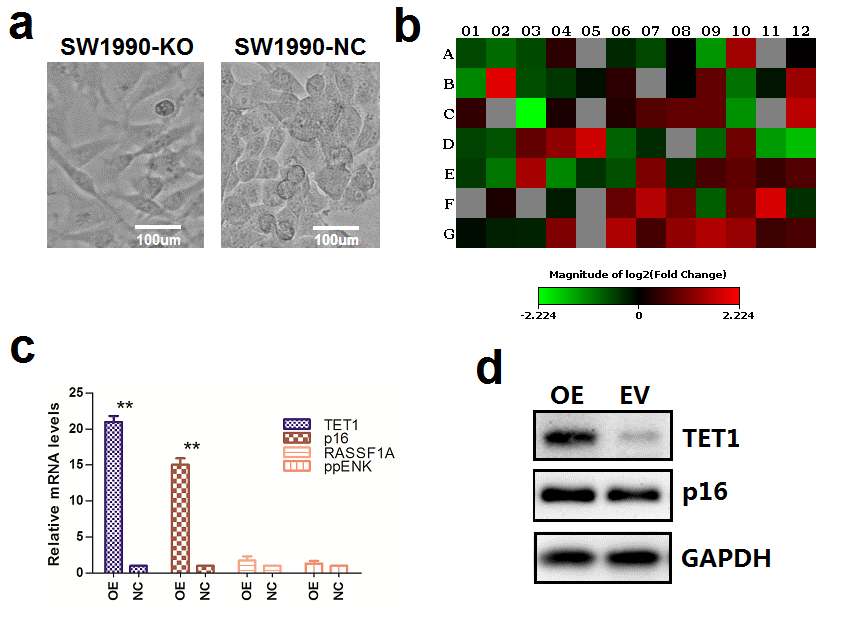

Supplement: Supplementary file 3 — : Figure S2 EMT changes in cells with TET1 knock-out & Pancreatic tumor suppressor genes in pancreatic cell lines. a morphology changes after TET1 knock-out in SW1990. b RNA array analysis of 84 EMT-associated genes in SW1990-KO and SW1990-NC cells, shown as a heatmap (log2-fold changes). c mRNA levels of p16, RASSF1A, and ppENK in BX-TET1-OE cells compared to NC cells, as detected by qRT-PCR. d Western blot of p16 in BX-TET1-OE cells compared to NC cells. (BMP 1552 kb) [file 13046_2019_1334_MOESM3_ESM.bmp]
